# Case Report: Optimum excision with split skin graft closure in the management of penile sebaceous carcinoma

**DOI:** 10.3389/fonc.2023.1095147

**Published:** 2023-07-26

**Authors:** Xeng Inn Fam, Chian Yong Liu, Suria Hayati Md Pauzi, Iqbal Hussain Rizuana

**Affiliations:** ^1^ Urology Unit, Department of Surgery, Universiti Kebangsaan Malaysia Medical Centre, Cheras, Kuala Lumpur, Malaysia; ^2^ Department of Anaesthesiology and Intensive Care, Universiti Kebangsaan Malaysia Medical Centre, Cheras, Kuala Lumpur, Malaysia; ^3^ Department of Pathology, Universiti Kebangsaan Malaysia Medical Centre, Cheras, Kuala Lumpur, Malaysia; ^4^ Department of Radiology, Universiti Kebangsaan Malaysia Medical Centre, Cheras, Kuala Lumpur, Malaysia

**Keywords:** sebaceous carcinoma (SC), penile ulcer, penile carcinoma, penile split skin graft, penile lesion

## Abstract

Sebaceous carcinoma (SC) is a rare malignant skin neoplasm derived from the meibomian gland of adnexal epithelium, which is frequently confused with basal cell carcinoma (BCC), exhibiting sebaceous differentiation and commonly found in the head and neck regions. We report a case of penile sebaceous carcinoma, an extremely rare anatomical site for SC. A 68-year-old man presented with a 4-month history of painless, non-healing ulcerated nodules over the left side of the penile shaft. Wedge biopsy showed adenocarcinoma with signet ring differentiation. We proceeded with wide local excision (WLE) of the lesion with the inclusion of the indurated skin and 5 mm of normal margin, followed by primary closure of the scrotal defect and split skin grafting of the penile shaft by using a thigh skin donor. The final histopathological examination revealed sebaceous carcinoma with pagetoid spread. The patient achieved recurrence-free survival without any form of adjuvant therapy after 4 years of follow-up.

## 1 Case report

### 1.1 Introduction

**Figure 1:**
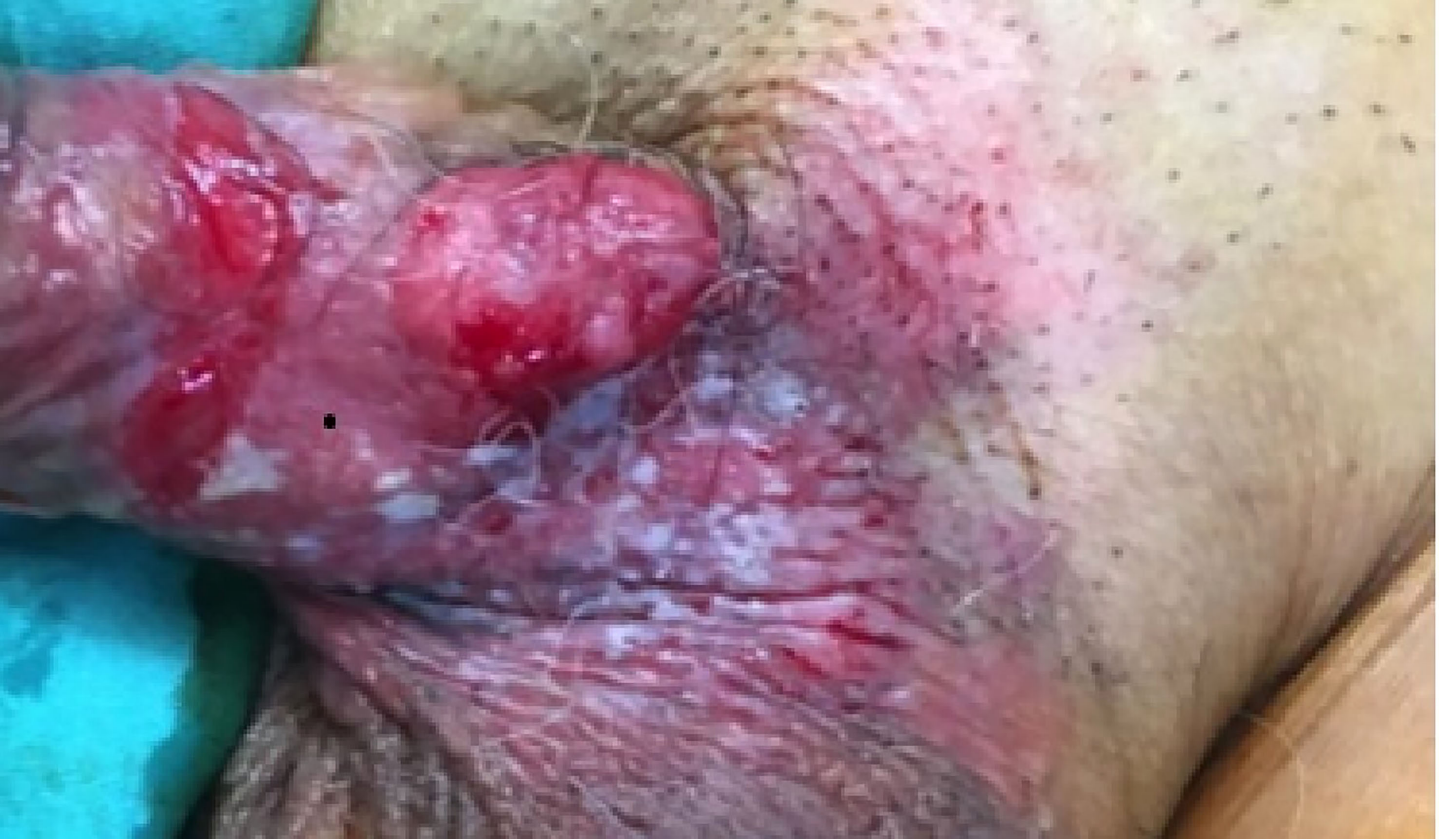
Ulcerative lesion measuring 20 × 15 × 10 mm located at left root of penis; surrounding penile skin and left hemiscrotum were indurated, inflamed, and haemorrhagic with whitish flaky deposits.

A 68-year-old man presented with a 4-month history of painless, non-healing ulcerated nodules over the left shaft of the penis. The lesion progressively increased in size with areas of induration extended from the left base of the penis to the scrotum, discharging non-foul-smelling haemoserous fluid. He was otherwise well without systemic manifestation. The patient was an active smoker with underlying hypertension. He had no family history of malignancy; had no previous exposure to chemicals, carcinogenic agents, or radiation; and denied a history of trauma, sexual promiscuity, or possible sexually transmitted disease. Clinically, the lesion measured 20 × 15 × 10 mm with ulceration at the root of the penis. The tumour was mobile. The surrounding penile skin and left hemiscrotum were indurated, inflamed, and haemorrhagic with whitish flaky deposits (**Figure 1**). Bilateral inguinal lymph nodes were not palpable. Systemic examinations were unremarkable.

Initial histopathological examination (HPE) of the biopsied lesion revealed a neoplasm characterized by signet ring morphology. A panel of immunohistochemical (IHC) studies showed positivity towards epithelial membrane antigen (EMA) and cytokeratin (CK) 7. There was a negative expression of tumour cells towards CK20, squamous markers (p63), and prostate-specific antigen (PSA). Based on the tumour morphology, the diagnosis of metastatic adenocarcinoma was considered. Screening for possible primary origin including tumour markers, cystoscopic examination, and upper and lower gastrointestinal endoscopic examination were unremarkable. Staging computed tomography (CT) scan of the thorax, abdomen, and pelvis was unremarkable. We proceeded with wide local excision (WLE) of the lesion with the inclusion of the indurated skin and 5 mm of normal skin margin. The scrotal defect was closed primarily; penile shaft defects were closed with split skin grafting (SSG) using a left thigh skin donor due to a big skin defect.

**Figure 2:**
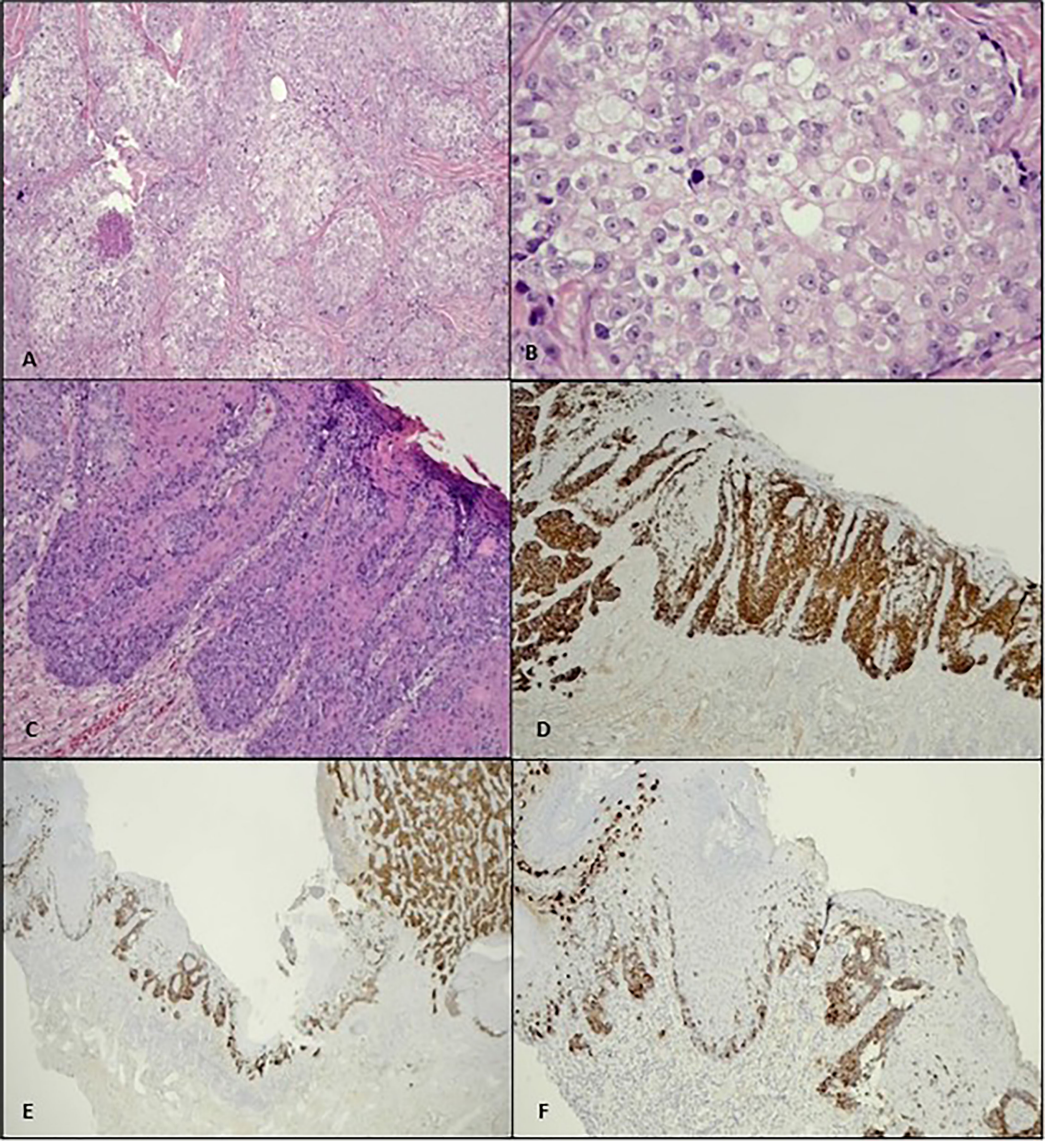
**(A)** Histologically, the penile tumour is composed of nests and lobules of malignant cells with clear foamy cytoplasm (H&E, ×10). **(B)** Higher power view of the tumour cells shows enlarged vesicular nuclei, conspicuous nucleoli, and abundant multivesicular cytoplasm (H&E, ×40). **(C)** Pagetoid spread along the dermo-epidermal junction is evident as adjacent to the main tumour mass (H&E, ×10), **(D)** which is highlighted by CK7 immunostain (×4). **(E, F)** Both the invasive and pagetoid components show positivity to EMA (×4, ×10).

HPE of the excised tumour nodule revealed malignant cells exhibiting sebaceous differentiation characterized by pleomorphic vesicular nuclei with prominent eosinophilic nucleoli, abundant vacuolated cytoplasm, and numerous mitotic figures (**Figures 2A, B**). Only a focal area of signet ring morphology was found. The malignant cells also showed pagetoid spread along the dermo-epidermal junction and surrounding the hair follicles (**Figure 2C**). IHC studies showed similar EMA and CK7 expression as in the biopsied sample (**Figure 2D**).

The tumour showed positivity to EMA and CK7, both in the invasive component and in the pagetoid spread (which is located within the epidermal and dermo-epidermal junction). **Figures 2E, F** show an EMA stain in this tumour. The malignant cells were negative for S100 and CK5/6. Considering the tumour morphology and IHC study, the final diagnosis of sebaceous carcinoma (SC) with pagetoid spread was made. The surgical margin was negative.

**Figure 3:**
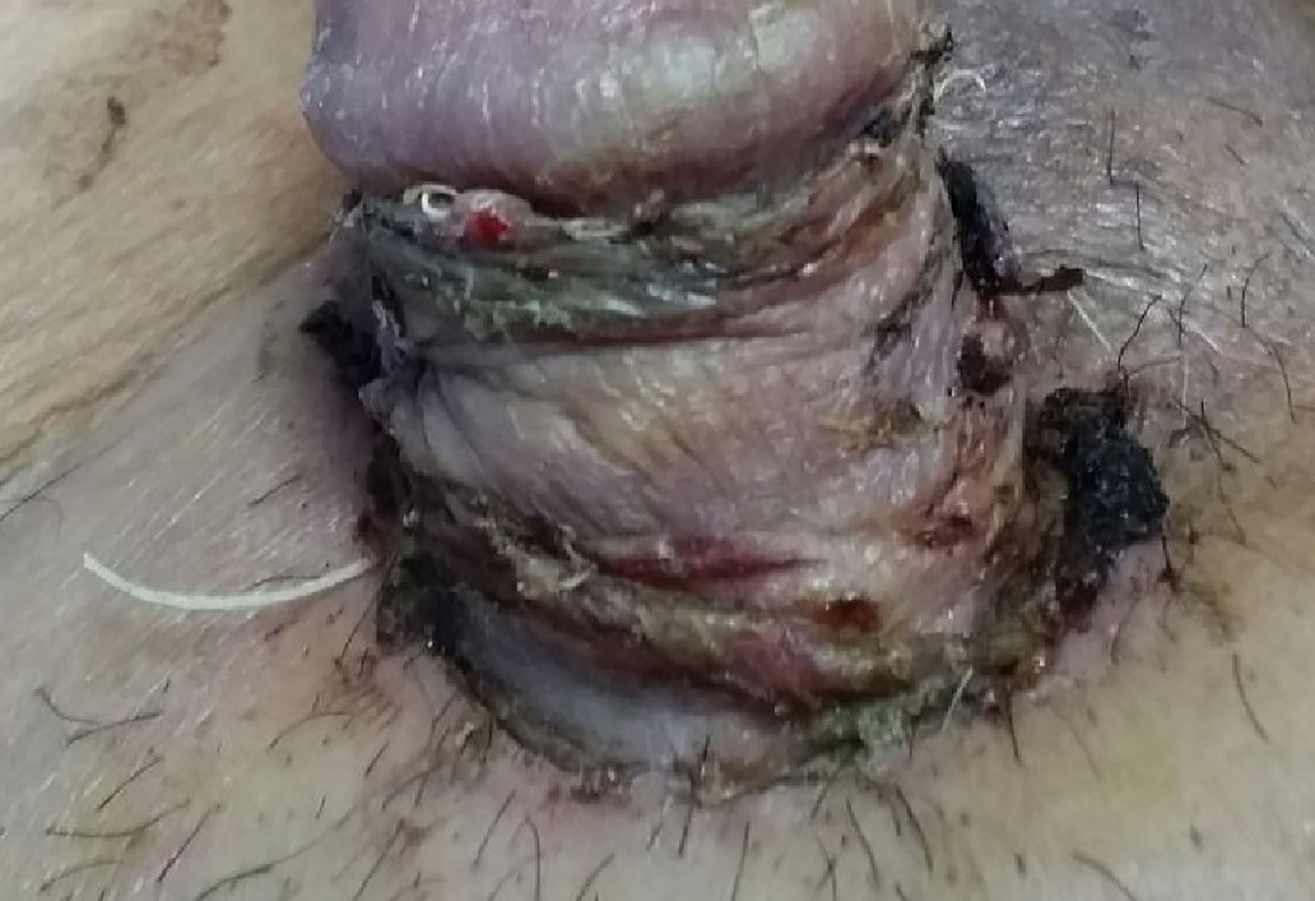
Post-op image showing split skin graft.

**Figure 4:**
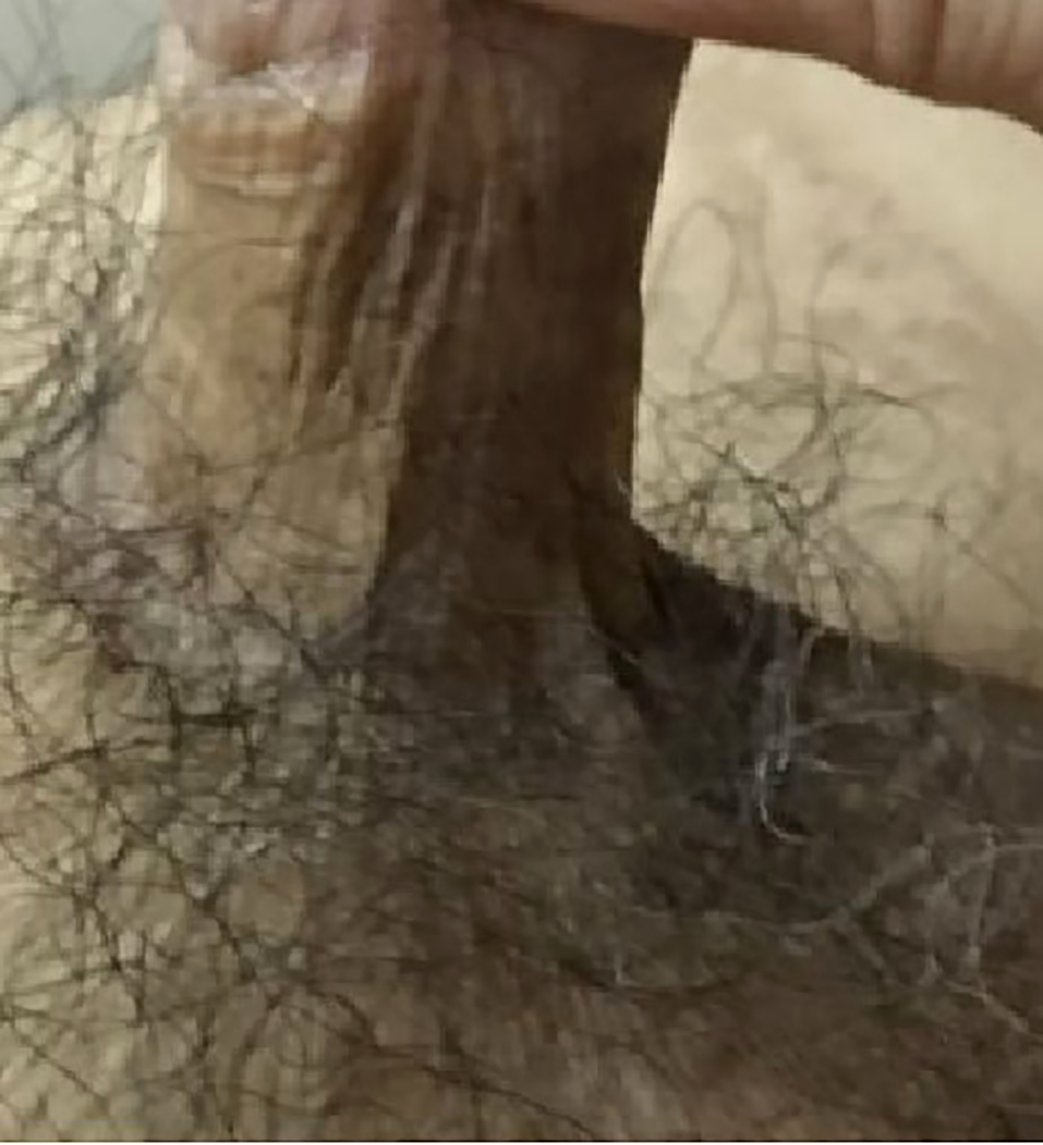
Split skin graft was fully taken by host without contracture; penis appeared normal.

Adjuvant radiotherapy and closed surveillance were discussed with the patient, and he preferred closed surveillance. SSG was completely taken by the host (**Figures 3**, **4**) with good penile erection.

He was followed up every 3 months for the first 2 years and 6 months after 2 years to assess for local recurrence and inguinal lymphadenopathy. An abdomen ultrasound was performed every 6 months. He did not show local recurrence, lymph nodes, or distance metastasis after 6 years of follow-up.

### 1.2 Discussion

Sebaceous gland carcinoma is a rare malignant skin neoplasm that constitutes fewer than 1% of cutaneous malignancies (1). It commonly occurs in the head and neck region, especially in the periorbital area (1, 2). Extraocular manifestation has been reported involving the external auditory canal, oral mucosa, scalp, vulva, ovaries, parotid, cervix, breasts, lungs, larynx, pharynx, palmoplantar region, nose, anal margin, penis, and salivary glands (3). Its incidence peaks in the seventh decade of life with a relative male:female ratio of 2:1 (4, 5).

Clinical and histopathological diagnoses at presentation can be challenging, as SC might resemble other benign and malignant epithelial neoplasms (2). It does not demonstrate any specific clinical features, leaving clinicians with diagnostic dilemmas, hence delaying diagnosis (5). Extraocular lesions had been described as slow-growing, firm, yellow-pink nodules, while some reported lesions had haemorrhagic surfaces or ulcers (5). The gold standard for diagnosis is tissue biopsy for HPE. Conventional microscopic findings of neoplastic cells with sebaceous differentiation on hematoxylin and eosin (H&E) staining are histologically diagnostic of SC. However, this histological recognition can be challenging, which requires adjunct IHC studies to differentiate it from other pathological entities (2, 5).

There are several differential diagnoses of SC from a histopathological point of view, which includes basal cell carcinoma (BCC) with sebaceous differentiation, clear cell squamous cell carcinoma, balloon cell melanoma, and metastatic clear cell renal cell carcinoma. BCC typically shows nests and lobules of basal cells with peripheral retraction artefacts and some mucinous stroma. Even though the basaloid cells may mimic germinative cells of SC, BCC does not show pagetoid intra-epidermal spread as was observed in this tumour. There were no retraction artefacts observed in this tumour. BCC is usually immunonegative to EMA while positive to BerEP4 (6).

Pagetoid cells are not specific to SC, as they can be seen in melanoma, extramammary Paget’s disease, and Paget’s disease of the nipple. Morphologically, they can look similar; therefore, immunohistochemistry studies are needed to differentiate them. Melanoma will be positive for S100 (our tumour is negative for S100). Both extramammary Paget’s and Paget’s of the nipple are positive for CK7. Nevertheless, Paget’s disease of the nipple usually demonstrates the presence of underlying ductal carcinoma *in situ* or invasive breast carcinoma. The invasive tumour in our case showed morphology that was consistent with SC. Pagetoid spread of carcinoma cells within the epidermis is a well-known phenomenon in SC (6).

Squamous cell carcinoma (SCC) may also show clear cell and signet ring features that may resemble SC. The clearing of cytoplasm (clear cell) and the peripherally located nuclei (signet ring pattern) give this tumour variant its name. Nevertheless, SCC is typically positive for CK5/6 immunostain, which is not present in this tumour (7).

Balloon cell melanoma is an uncommon variant of malignant melanoma, which is characterized by the ballooning of the neoplastic melanocytes, which resemble pseudolipoblast. The neoplastic cells may show melanin pigments within the cytoplasm. Immunohistochemical studies play an important role in delineating the origin of these balloon cells, as the neoplastic melanocytes are immunopositive to S100, HMB45, Melan A, and SOX 10 (8). In our case, the S100 stain was negative.

Clear cell renal cell carcinoma (CcRCC) is the most common type of renal cell carcinoma (RCC), comprising up to 70% of all RCC cases. The accumulation of lipids and glycogen gives the cytoplasm of the neoplastic cells its clear morphology. Metastasis is common in RCC and therefore needs to be excluded in any tumour with clear cell morphology. CcRCC typically shows a delicate capillary network within the tumour, which is not observed in our case. CcRCC will show immunopositivity to CD10, CA-IX, PAX8, and RCC markers while immunonegativity to CK7 (9). The neoplastic cells in this case show strong positivity to CK7. There was also no history of renal tumours in this patient.

In 1974, Rulon and Helwig addressed the confusing nomenclature and systematically classified sebaceous gland tumours into three categories: sebaceous adenoma, BCC with sebaceous differentiation, and sebaceous carcinoma (4). SC was described as the presence of cytoplasmic vacuolization and positivity for lipid stains of the well-differentiated tumoural cells such as the oil red 0 or Sudan IV techniques, separating it from basosebaceous epithelioma (4). The clinical significance in differentiating these two lies in the biological property of the tumour whereby basosebaceous epithelioma follows the more indolent behaviour of BCC, rendering slow progression, infrequent recurrence, and rare metastasis, while SC is rather aggressive with a high risk of regional metastases (4).

Earlier reports recommended WLE and removal of grossly involved lymph node chains for extraocular SC (4, 10, 11). Data for analysis on prophylactic lymphadenectomy were limited (4). Furthermore, opinions were divided on postoperative irradiation and chemotherapy. Adjuvant therapy was employed in most cases with disseminated disease; hence, the benefit recorded may not be reflective of tumour sensitivity to irradiation or chemotherapeutic agents.

According to Yamamoto et al., although there is a role for sentinel lymph node biopsy (SNB) for intraocular SC, they did not recommend routine SNB for extraocular SC except for vulvar cancer. Other than SC vulva, which has established systematic review and meta-analysis, there are limited data for the rest of extraocular SC. Due to scarce information on SNB for penile sebaceous carcinoma well as its value, we did not proceed with SNB in our patient.

The current standard practice of management for localized SC is WLE with a tumour-free margin of 5–6 mm, followed by radiotherapy (1). We achieved good negative margin excision for this patient, which left behind a big penile skin defect that was impossible for primary closure. We closed the penile defect with SSG from the left tight based on our experiences in managing penile lymphogranuloma (6). The patient achieved 4 years of recurrence-free survival with optimum tumour margin excision without any form of adjuvant therapy. SSG uptake was excellent with good penile erection. Diagnosis and treatment in the early stage of the disease were strong factors for his excellent prognosis. Metastatic sebaceous carcinoma is very rare, with very little evidence on the role of chemotherapy in the treatment of metastatic disease (1).

### 1.3 Conclusion

Clinical and histopathological diagnoses of SC can be challenging. WLE with a good tumour margin is important to achieve good recurrence-free survival.

## 2 Limitation

The rarity of sebaceous carcinoma of the penis makes the diagnosis of SC penis difficult. There is limited literature available, which is mostly case reports and case series. The lack of prospective studies and standard treatments increased the challenges faced in this case.

## 3 Patient’s perspective

The patient was worried and anxious about his diagnosis during the initial stages. However, with adequate discussion and counselling by multidisciplinary teams (urology and oncology), the patient understood his condition and the operative procedure. He became hopeful and agreed to proceed with his treatment.

## Data availability statement

The original contributions presented in the study are included in the article/supplementary material. Further inquiries can be directed to the corresponding author.

## Ethics statement

Written informed consent was obtained from the patient for the publication of his case and associated images.

## Author contributions

XF: conceptualized the article, prepared the draft, and edited and finalized the draft. IR: manuscript preparation, review and editing. CL: anaesthesiologist, resources. SP: pathologist, prepared the slides. All authors contributed to the article and approved the submitted version.
